# Primary Leiomyosarcoma of the Adrenal Gland: A Case Report with Immunohistochemical Study and Literature Review

**DOI:** 10.1155/2014/489630

**Published:** 2014-03-04

**Authors:** Murat Tolga Gulpinar, Asif Yildirim, Berrin Gucluer, Ramazan Gokhan Atis, Cengiz Canakci, Cenk Gurbuz, Turhan Caskurlu

**Affiliations:** ^1^Department of Urology, Istanbul Medeniyet University Goztepe Training and Research Hospital, Doktor Erkin Caddesi, Kadikoy, 34722 Istanbul, Turkey; ^2^Department of Pathology, Istanbul Medeniyet University Goztepe Training and Research Hospital, Doktor Erkin Caddesi, Kadikoy, 34722 Istanbul, Turkey

## Abstract

Primary adrenal leiomyosarcoma is extremely rare tumor. We report a case with adrenal leiomyosarcoma. Our case was a 48-year-old man who presented with lower urinary tract symptoms. Ultrasonography and magnetic resonance imaging revealed approximately 9 cm solid mass originating from right adrenal gland. He underwent right adrenalectomy. Pathology of the specimen showed histologic and immunohistochemical features of adrenal leiomyosarcoma.

## 1. Introduction

Primary leiomyosarcoma of the adrenal gland is a malignant tumor very rarely encountered in the literature and is without any specific findings by laboratory tests and radiologic imaging modalities. Herein, we present a rare case of primary leiomyosarcoma of the right adrenal gland in a 48-year-old male patient who presented with lower urinary tract symptoms. We aim to present a case with a definitive diagnosis of adrenal leiomyosarcoma, which was histopathologically established based on the microscopic examination and immunohistochemical staining of the specimen from the time of diagnosis to its treatment. A review of the recent literature is also presented.

## 2. Case

**Figure 1:**
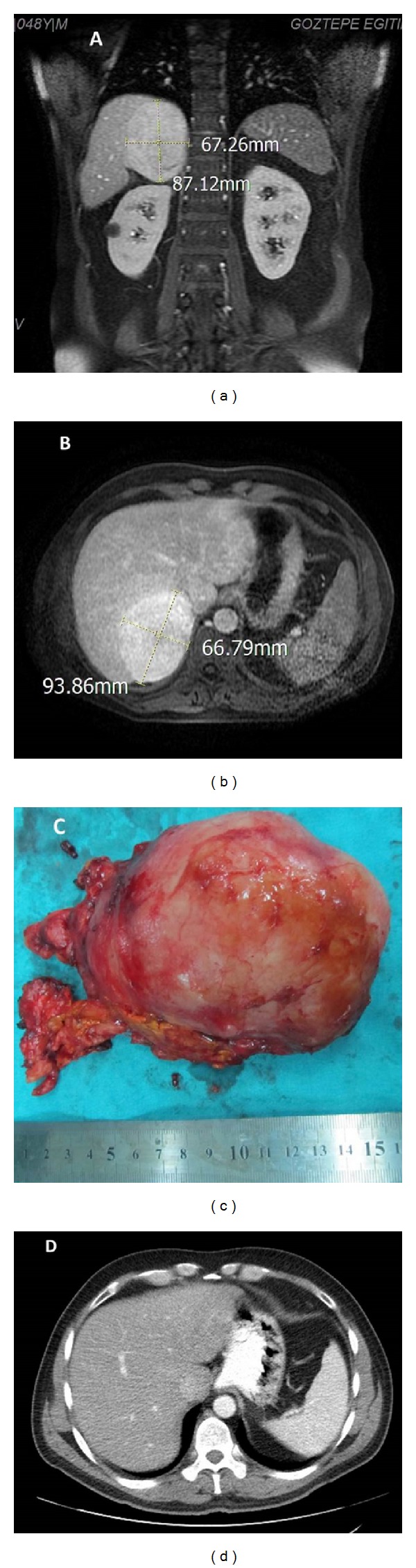
Magnetic resonance images of a 9 cm right adrenal leiomyosarcoma. T1-weighted images in coronal section (a) and transverse section show a homogenous solid mass in the right suprarenal area. (c) Macroscopic appearance of roundish, fibrous neoplasia. (d) Six months after right adrenalectomy, postoperative abdominal computerized tomography demonstrated no signs of local recurrence.

A 48-year-old male patient consulted an outpatient urology clinic with complaints of frequent urination and awaking during night hours due to the need to urinate. During his physical examination, a mass was palpated on the upper right abdominal quadrant. Upon ultrasonography, a right adrenal mass measuring 86 × 83 mm was detected, and the patient was hospitalized for further investigation and treatment. The mass was evaluated by the endocrinology department for identification. Upon abdominal magnetic resonance imaging (MRI), a 90 × 65 mm hypervascular solid mass originating from the right adrenal gland and extending to the right hepatic lobe was observed, with intense contrast uptake on postcontrast images (Figures 1(a) and 1(b)).

The rectal examination findings and PSA values of the patient, whose father had been diagnosed with prostatic carcinoma, were within the normal limits. A routine laboratory examination and chest X-ray findings were unremarkable. To evaluate the endocrine activity of the mass, the 24-hour vanyl-mandelic acid, metanephrine, and normetanephrine levels in the urine and the urinary free cortisol levels were measured; all results were within the normal limits. As the patient was not hypertensive, aldosterone and renin levels were not determined, and the lack of relevant clinical findings rendered the measurement of sex steroids useless. In light of these findings, laparoscopic adrenalectomy was planned for the patient. Bleeding foci blurred the vision during laparoscopic dissection; thus, we switched to the open adrenalectomy technique. The mass, which had not infiltrated into adjacent structures, was completely dissected, and the specimen was sent for histopathological examination (Figure 1(c)).

**Figure 2:**
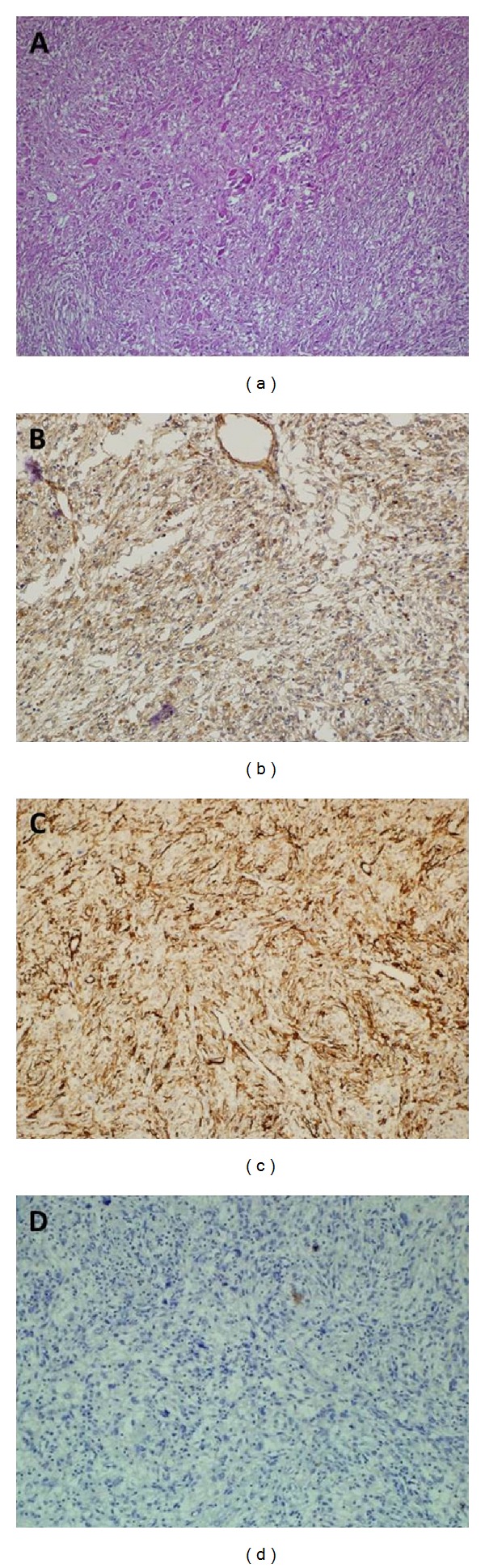
(a) Microscopic details of tumor—infiltration of inflammatory cells and spindle-shaped neoplastic cells (H and E, ×100). (b) Positive Smooth Muscle Actin (SMA) immunostaining (×100). (c) Positive Caldesmon immunostaining (×100). (d) Negative Anaplastic Lymphoma Kinase (ALK) immunostaining (×200).

The postoperative period was uneventful, and the patient was discharged with due recommendations on the 3rd postoperative day. The adrenal mass had dimensions of 110 × 80 × 65 mm and weighed 370 grams. Serial cuts revealed a yellow-creamy white solid mass with a central necrotic area. Sections obtained from the mass with a thick fibrous capsule disclosed the development of spindle cells associated with predominantly diffuse plasma cell infiltration prevalent in the necrotic areas. Neoplastic cells characterized by occasional bizarre giant cells, widespread apoptosis, and patchy areas of mitosis were observed. In consideration of the location of the tumor, the differential diagnosis was expanded to include tumors of the adrenal cortex and medulla, primary tumors of the liver, and inflammatory myofibroblastic tumors; we then proceeded with immunohistochemical staining. Immunohistochemical analyses of the neoplastic cells, including pancytokeratin, inhibin, synaptophysin, chromagranin, CD30, CD31, CD34, CD117, S100, ALK, myogenin, lambda, and kappa light chain proteins yielded predominantly immunonegative results. In contrast, vimentin demonstrated strongly diffuse and SMA moderately diffuse immunopositivity, and Ki67 demonstrated 4% immunopositivity. In light of these findings, a diagnosis of low-grade leiomyosarcoma was made (Figure 2).

The patient, whose adrenal mass lesion without any sign of infiltration was completely removed in compliance with the principles of radical surgery, did not receive any adjuvant therapy. He did not encounter any medical problem during his 8-month-long follow-up period, and abdominal CT performed at the 6th postoperative month did not reveal any evidence of recurrence or metastatic mass lesion (Figure 1(d)).

## 3. Discussion

Although leiomyosarcomas are the most frequently encountered type among intraabdominal soft tissue malignancies, only 13 cases of primary leiomyosarcoma of the adrenal gland have been reported to date. To our knowledge, our patient constitutes the 14th case [1, 2]. Kanthan et al. reported and retrospectively analyzed 94 cases of adrenal incidentaloma, which included only one case of primary adrenal leiomyosarcoma [3]. Although the etiologies of these rare tumors have not been clearly elucidated, a few studies have suggested etiologic roles of HIV and Epstein Barr virus [4]. Nonetheless, very scarce information is available on the mechanisms underlying the aggressive behavior of these tumors. Chromosomal aberrations might contribute to the morphological transformation of leiomyosarcomas [5, 6], and radical surgical resection of these tumors appears to be the only treatment modality. The histologic grade is correlated with its biological behavior and prognosis [7, 8], and these tumors rarely metastasize to regional lymph nodes, with metastases most frequently observed in the lungs and liver. Postoperative adjuvant radiotherapy is recommended for locally advanced disease [9]. However, the effectiveness of chemotherapy is very limited and can be used in cases of inoperable tumors, incomplete resections, and metastatic lesions. Although the prognosis for patients with leiomyosarcomas cannot be currently predicted, venous thrombosis, neighboring organ invasion, and distant metastases are indicators of a relatively worse prognosis [10]. Lujan and Hoang reported that patients with metastatic adrenal leiomyosarcomas died shortly after adrenalectomy. The longest reported survival time is 21 months [1], and survival rates are associated with the dimensions of the tumor, the applicability of radical surgical resection, and the morphologic grade of the tumor [11, 12].

In conclusion, our case was evaluated as a histopathologically low-grade leiomyosarcoma without any evidence of invasion into any adjacent tissue or distant metastasis, constituting a lesion that can be excised with an intact and safe surgical margin in compliance with the principles of radical surgery. Although optimal treatment of advanced adrenal leiomyosarcoma may be accepted as complete resection followed by a combination of radiotherapy and chemotherapy, radical surgery without adjunctive therapies may provide oncological control in low-grade and localized cases.
